# F. R. McManus, M. D.

**Published:** 1885-04

**Authors:** 


### F. R. McMANUS, M. D.

We regret to have to announce the death of Dr. F. R. McManus, which occurred at Baltimore March 2d, 1885. Dr. McManus was one of the "pioneer homœopathists," alas, now so few in number. After practicing allopathy for some years, he became converted to true therapeutics as taught by Hahnemann, in 1837, and has been an able and strict follower of the master since that date.

